# Human Body Rhythms in the Development of Non-Invasive Methods of Closed-Loop Adaptive Neurostimulation

**DOI:** 10.3390/jpm11050437

**Published:** 2021-05-20

**Authors:** Alexander Fedotchev, Sergey Parin, Sofia Polevaya, Anna Zemlianaia

**Affiliations:** 1 Institute of Cell Biophysics, Russian Academy of Sciences, 3 Institutskaya St., Pushchino, 142290 Moscow Region, Russia; 2 Lobachevsky State University of Nizhni Novgorod, 23 Prospekt Gagarina, 603950 Nizhny Novgorod, Russia; parins@mail.ru (S.P.); s453383@mail.ru (S.P.); 3 Moscow Research Institute of Psychiatry, Branch of the Serbsky’ National Medical Research Center of Psychiatry and Narcology, Russian Ministry of Health, 3 Poteshnaya St., 107076 Moscow, Russia; a_zemlyanaya@mail.ru

**Keywords:** non-invasive brain stimulation, closed-loop adaptive sensory stimulation, endogenous rhythms, respiratory rate, heart rate, electroencephalogram (EEG) rhythms, automatic modulation, correction of functional state

## Abstract

The creation and improvement of non-invasive closed-loop brain stimulation technologies represent an exciting and rapidly expanding field of neuroscience. To identify the appropriate way to close the feedback loop in adaptive neurostimulation procedures, it was previously proposed to use on-line automatic sensory stimulation with the parameters modulated by the patient’s own rhythmical processes, such as respiratory rate, heart rate, and electroencephalogram (EEG) rhythms. The current paper aims to analyze several recent studies demonstrating further development in this line of research. The advantages of using automatic closed-loop feedback from human endogenous rhythms in non-invasive adaptive neurostimulation procedures have been demonstrated for relaxation assistance, for the correction of stress-induced functional disturbances, for anxiety management, and for the cognitive rehabilitation of an individual. Several distinctive features of the approach are noted to delineate its further development.

## 1. Introduction

Non-invasive brain stimulation techniques, including transcranial magnetic stimulation, transcranial direct current stimulation, and various kinds of sensory stimulation, are successfully used as therapeutic tools in psychiatry and neurology and are also applied in cognitive neuroscience to study the functioning of the brain [1]. For example, non-invasive brain stimulation is increasingly used as a clinical intervention for neuropsychiatric disorders [2,3] as a method to understand the neural mechanisms underlying cognition [4] and as the tool to enhance cognitive rehabilitation after stroke [5]. Sensory brain stimulation with various modalities, such as tactile, auditory, and visual stimulation, can be effectively used in brain–computer interface systems to provide paralyzed patients with an alternative communication channel [6]. The combination of transcranial magnetic stimulation with electroencephalography allows for non-invasive investigation of cortical response and connectivity in the human cortex and can be used to study changes in cortical connectivity and signal propagation from healthy to pathological brains [7].

The first generation of brain stimulation systems used an open loop fashion where stimulation parameters (e.g., amplitude, duration, and frequency) remain fixed over time and are not responsive to any real-time physiologic variables [8]. However, the physiology of the nervous system or the modulated organ can be dynamic, and the same stimulus may have different effects depending on the underlying state. As a result, open-loop stimulation may fail to restore the desired function or cause side effects [9].

## 2. Benefits of Closed-Loop Systems

Higher-efficacy, second-generation brain stimulation techniques (closed-loop systems) can modulate or adapt the therapeutic stimulation output by responding in real time to the local physiologic environment [10]. In closed-loop therapy the stimulation is adjusted by a device or algorithm in response to changes in the patient’s electrical brain activity and may provide more precise and patient-specific treatments [11]. A closed-loop stimulation device might deliver stimulation more proficiently by performing stimulation only when brain function is damaged or shows abnormal neural activity and synchronizing each stimulus with the patient’s instantaneous brain state [12]. Such closed-loop neurostimulation therapy may offer advantages over open-loop therapies by increasing the efficacy of stimulation, improving the clinical benefit of stimulation, and reducing the side effects of stimulation [13].

For example, it was shown that the brain-actuated functional electrical stimulation elicits significant, clinically relevant, and lasting motor recovery in chronic stroke survivors due to the involvement of mechanisms of functional neuroplasticity [14]. Closed-loop vibration stimulation could effectively influence heart rhythm and stabilize the autonomic nervous system [15]. There are a number of studies demonstrating the advantages of closed-loop electroencephalography (EEG) utilization for human cognitive engagement and regulation of arousal to improve task performance. For example, an EEG-based closed-loop system has been recently developed to increase user engagement through a continuous pursuit task and associated training paradigm [16]. The EEG-based closed-loop brain–computer interface is shown to induce dynamically shifting arousal to affect online task performance [17].

It is important to note that closed-loop sensory stimulation is also used in neurofeedback training, where subjects are fed back sensory information about some measure of their brain activity that they are instructed to modify [18]. However, the efficacy of neurofeedback training is limited by several factors, including the correct decoding of human thoughts and application of effective learning strategies [19]. To reduce these limitations, automatic adaptive stimulation architectures that can dynamically accommodate the transient nature of brain features should be used. The closed-loop neurostimulation methodology for which stimulation parameters are automatically adapted by biomarker feedback can streamline the individualization process of these treatments. The choice of biomarkers suitable for informing stimulation parameters has emerged as a primary developmental goal for closed-loop technologies [20].

## 3. Human Endogenous Rhythms as Modulating Factor for Sensory Stimulation

In order to identify the appropriate way to close the feedback loop in adaptive neurostimulation procedures, Fedotchev 1996 [21] and Salansky et al. 1998 [22] previously proposed to use on-line automatic sensory stimulation with the parameters modulated by the patient’s own rhythmical processes, such as respiratory rate, heart rate, and electroencephalogram (EEG) rhythms. These rhythmical processes are closely interrelated and form the basis for homeostatic constancy, efficiency of physiological processes, and the adaptation to internal/external changes and requirements [23], participate in rhythmic facilitation of sensory processing [24], and play a vital role in recovering the neural plasticity and training or regulating brain activities [25]. Importantly, these rhythmic processes are the sources of interoceptive signals vital for the emotional sphere of a person [26].

The clinic-like testing of the approach proposed in [21] was carried out on a model of analgesic electroneurostimulation with the automatic control of the parameters of the stimulating current by the patient’s breathing rate. In total, 12 volunteers suffering from different forms of etiology pain participated in the study. It was shown that the most significant changes after only a single treatment procedure occurred in the level of subjective pain ratings, which dropped by half. A significant pain reduction was accompanied by the relaxation of the central nervous system (deepening of respiration and reduction of muscular tension) and positive shifts in the patients’ self-assessments of well-being and mood [21].

The goal of the present study was to assess the state of the art in this specific line of research, where the parameters of closed-loop sensory stimulation are automatically modulated by human body rhythmical processes. Literature searches were conducted using OVID (Medline, Health Star, Embase + Embase Classic) and PubMed databases.

## 4. Recently Developed Methods of Closed-Loop Adaptive Neurostimulation

To date, the advantages of using automatic closed-loop feedback from human endogenous rhythms for non-invasive adaptive neurostimulation have been demonstrated in several studies (Table 1).

Positive relaxation effects were achieved through complex acoustic stimulation automatically modulated by the patient’s current values of heart rate variability [31]. The possibility to augment endogenous slow-wave oscillations in humans by closed-loop transcranial alternating current stimulation with the aim of improving consolidation of recent experiences into long-term memory has been shown [34]. The presentation of acoustic stimuli on-line generated by the software-guided transformation of a subject’s dominant EEG rhythm was recently shown to induce clinically significant decrease in post-traumatic stress symptoms [30] and improvements in heart rate variability, baroreflex sensitivity, and sleep [32]. The authors came to the conclusion that rapid updating regarding its own pattern, as well as resonance between the audible tones and oscillating brain networks, provides the brain a chance to auto-calibrate, self-adjust, “relax”, and reset/get unstuck from what have been persisting stress/trauma response patterns [38].

Literature data show that the most popular way to form the feedback from human endogenous rhythms is its software-guided transformation into music or music-like stimuli. The experimental basis for this line of research involves the numerous data showing that fluctuations in the electrical activity of the brain are able to synchronize with the temporal patterns of external influences and lead to the therapeutic effect of music on cognitive or motor symptoms [40]. In addition, musical stimulation is known to have a number of cognitive, psychosocial, and behavioral benefits, especially for people with neurological disorders, providing a basis for the development of non-drug therapies [41].

For example, it is shown that the successful correction of the functional state in patients with movement disorders can be achieved as a result of presentations of music-like stimuli automatically generated by transforming the alpha or mu EEG rhythms [29]. The human emotional state can be effectively corrected by presentations of music with harmonic, rhythmic, and timbre components automatically synthesized by computer transformations of theta, alpha, beta, and gamma EEG rhythms [36]. An auditory interface for the detection and treatment of anxiety in children has been developed [28]. The interface, called “Biomusic”, maps physiological signals to music (e.g., electrodermal activity to melody; skin temperature to musical key; heart rate to drumbeat; and respiration to a “whooshing” embellishment resembling the sound of an exhalation). The authors argue that the technology holds promise as a biofeedback system for anxiety management.

In our initial studies, we used presentations of classical music automatically modulated by the current amplitude of theta, alpha, and beta EEG rhythms. A method of musical neurofeedback, which combines the maximal personalization of neurofeedback with an unconscious perception of music therapy, has been developed and tested [27]. A group of pregnant women suffering from stress-induced complications of pregnancy participated in the study. Patients took a comfortable position in an armchair or on a couch, and their eyes were closed. Quiet music served as a feedback signal. Each patient chose a specific type of musical composition during the first examination from a list offered. Classical music was presented only when the patient (using a special individual strategy to attain the necessary degree of relaxation) was able to change a given EEG rhythm in the desired manner. During treatment sessions, the occipital EEG was recorded and processed on-line to measure the current amplitudes of the theta, alpha, and beta EEG rhythms. The task of the patient was to feel, realize, and remember her own sensations when hearing the music so that the music would not stop.

It was found [27] that the patients could learn to voluntarily control their own EEG activity via musical neurofeedback. Questioning and testing of patients revealed their positive attitude to training sessions, a decrease in the stress level, and positive changes in their psycho-emotional status. However, the effectiveness of training the patients to control their own EEG rhythms was relatively low. This was attributed to a high heterogeneity of traditional EEG rhythms used in the study. As it is known from the literature, each traditional EEG rhythm is not a unitary phenomenon; rather, it is comprised of different oscillations with different frequencies across a broad range [42]. Therefore, the use of traditional EEG rhythms in neurofeedback procedures can be compared to playing the piano in mittens—trying to press the right keys, the pianist will inevitably also play adjacent ones and cause a cacophony of sounds. It was concluded that it is necessary to use narrow-frequency spectral components of the EEG (EEG oscillators), which are significant for the subject, instead of predetermined, excessively wide-frequency traditional EEG rhythms (theta, alpha, beta, etc.) [27].

Later, to correct stress-induced states, we developed an original music-based approach. The approach is named “Music of the Brain” and consists of musical or music-like stimulation on-line controlled by discrete components of subject’s EEG (EEG oscillators). By EEG oscillator of a patient is meant the narrow-frequency part of the subject’s EEG that is meaningful and significant to him. An original method of EEG processing has been developed to reveal the EEG oscillators of the subject [33]. The method employs a fast Fourier transform procedure on short (4–5 s) periods of background EEG recordings that are sequentially shifted relative to each other with a 50% overlap. The level of noise is suppressed by selecting only the most pronounced spectral peaks. When such spectral peaks are sequentially accumulated for the whole period of background EEG recording, the resulting spectrum is based on the summation of a large number of individual short-term spectra. It has high (0.2–0.25 Hz) frequency resolution and provides information on stable and specific narrow-band EEG oscillators that are important for the subject. It was shown that, with presentations of music, automatically controlled by EEG oscillators of the patient, a decrease in the stress levels, normalization of the EEG, and positive shifts in the psycho-emotional statuses of human subjects are observed [33,35].

Recently, we assumed that the effectiveness of music-like stimulation can be increased if it is automatically controlled not only by the patient’s EEG oscillators but also by their heart rate. Indeed, the biopotentials of the brain and the heart are a source of interoceptive signals that play an important role in maintaining the optimal physical, emotional, and mental health of a person [43,44], and their use in treatment procedures is a roadmap for the development of neurotechnologies [45]. Based on these considerations, a neurointerface was developed in which the on-line registered amplitude of the subject’s EEG oscillator was automatically converted into music-like signals resembling flute sounds with smooth variations in pitch and intensity. These EEG-based music-like stimuli were supplemented with weak auditory signals corresponding to the subject’s heart rate. Simultaneously, LED stimuli generated on the basis of subject’s native EEG were presented to stressed volunteers. An increase in the alpha EEG power relative to the background, as well as positive emotional reactions and significant shifts in the indicators of functional state, were observed after just a single treatment procedure [37].

In an attempt to reveal possible mechanisms of the treatments, recently, we compared the effects observed in subjects under the light and music stimulation modulated by their own brain and heart biopotentials with the effects of the same stimulation modulated by the biopotentials of another person. A significant increase in the power of the main EEG rhythms, accompanied by significant positive changes in psychophysiological indicators and emotional responses to stimulation, was observed only under light and music stimulation controlled by the subject’s own brain and heart biopotentials. These data are attributable to the integration of perception and processing of interoceptive signals significant for humans into the resonance mechanisms of the central nervous system that provide the normalization of functional state due to stimulation [39].

## 5. Conclusions

The described literature data clearly indicate that human endogenous rhythms—the respiratory rate, the heart rate, and EEG rhythms—can be successfully used to develop non-invasive methods of closed-loop sensory stimulation for the effective correction of functional disturbances and the cognitive rehabilitation of an individual. The proposed approach has several distinctive features:

– High personalization through the use of closed-loop feedback from the patient’s own bioelectric characteristics;
– Involvement of interoceptive signals in the mechanisms of multisensory integration, neuroplasticity, and resonance mechanisms of the brain;
– Automatic operation, without conscious efforts of an individual, and control of therapeutic sensory stimulation, which makes it possible to use adaptive neurostimulation to correct functional disturbances in patients with altered levels of consciousness independently from their motivation.

The discussed approach appears to be a promising way to develop new effective methods for the timely correction of human functional disorders. The most promising seems to be the utilization of complex multimodal feedback from several rhythmic processes of the patient, including the heart rate, the respiratory rate, and human EEG oscillators. This could make treatment interventions more personalized and effective.

## Figures and Tables

**Table 1:** Successful application of human endogenous rhythms as a modulating factor in closed-loop adaptive neurostimulation paradigms for the correction of functional disturbances.

Condition	Stimulation	Modulating Rhythm	Reference
Musculoskeletal pain reduction	Electrical stimuli	Breathing rate	Fedotchev 1996 [21]
Correction of functional disturbances during pregnancy	Classical music	Theta, alpha, beta EEG rhythms	Fedotchev, Kim 2006 [27]
Anxiety reduction	Music-like stimuli	Heart rate, breathing rate	Cheung et al. 2016 [28]
Treatment of movement disorders	Music-like stimuli	Alpha or mu EEG rhythms	Deuel et al. 2017 [29]
Post-traumatic stress reduction	Acoustic stimuli	Selected EEG frequencies	Tegeler et al. 2017 [30]
Relaxation assistance	Music-like stimuli	Heart rate	Yu et al. 2018 [31]
Remediation of health concerns	Acoustic stimuli	Selected EEG frequencies	Shaltout et al. 2018 [32]
Health protection	Music-like stimuli	Alpha-EEG oscillator	Fedotchev et al. 2018 [33]
Improving consolidation of recent experiences into long-term memory	Transcranial alternating current stimulation	Endogenous slow-wave oscillations	Ketz et al. 2018 [34]
Stress-induced state correction	Classical music	Alpha-EEG oscillator	Fedotchev 2018 [35]
Emotional state correction	Music-like stimuli	Theta, alpha, beta, gamma EEG rhythms	Ehrlich et al. 2019 [36]
Stress-induced state correction	Music-like stimuli + photic stimuli	Alpha-EEG oscillator + heart rate + native EEG	Fedotchev et al. 2019 [37]
Stress-related symptom reduction	Acoustic stimuli	Selected EEG frequencies	Tegeler et al. 2020 [38]
Stress-induced state correction	Music-like stimuli + photic stimuli	Alpha-EEG oscillator + heart rate + native EEG	Fedotchev et al. 2020 [39]
